# Developing and Implementing the Massachusetts Comprehensive Cancer Control Coalition Survivorship Summit

**Published:** 2009-12-15

**Authors:** Stephenie C. Lemon, Marianne N. Prout, Junaidah B. Barnett, Maureen Sullivan Flynn

**Affiliations:** Division of Preventive and Behavioral Medicine, University of Massachusetts Medical School. Dr Lemon is also a member of the Survivorship Working Group of the Massachusetts Comprehensive Cancer Control Coalition.; Boston University School of Public Health, Boston, Massachusetts. Prout is also a member of the Survivorship Working Group of the Massachusetts Comprehensive Cancer Control Coalition.; Tufts University School of Medicine and the Friedman School of Nutrition Science and Policy at Tufts University, Boston, Massachusetts. Barnett is also a member of the Survivorship Working Group of the Massachusetts Comprehensive Cancer Control Coalition.; Dana Farber Cancer Institute, Lance Armstrong Foundation Adult Survivorship Clinic, Boston, Massachusetts. Flynn is member of the Survivorship Working Group of the Massachusetts Comprehensive Cancer Control Coalition.

## Abstract

Cancer survivors face numerous medical and psychosocial challenges, which the medical and public health systems are ill-equipped to deal with. In May 2008, the Massachusetts Comprehensive Cancer Control Coalition conducted a Survivorship Summit to elicit input from cancer survivors and professionals on developing system-level action plans for cancer survivorship issues. We describe how health care and public health professionals can implement similar events. Our results suggest that a cancer survivorship summit can be a valuable tool for cancer coalitions and advocacy organizations in determining survivorship agendas and action plans.

## Introduction

More people than ever are surviving a cancer diagnosis. Almost 12 million cancer survivors live in the United States, and that number is expected to increase given recent trends (1). Despite the decrease in deaths, many cancer survivors face medical and psychosocial problems as a result of the cancer they have survived or the treatment they received (2). The current health care and public health systems are not designed to handle the transition from active treatment to posttreatment care (3,4). In its report *From Cancer Patient to Cancer Survivor: Lost in Transition,* the Institute of Medicine addresses the medical and psychosocial issues faced by cancer survivors and makes recommendations for improving the health and quality of life of cancer survivors. Cancer survivors face medical and psychosocial late effects (eg, interpersonal consequences, fatigue, pain, recurrence, depression and anxiety, distress, work and financial problems) (5-10). Such problems are difficult to address from a public health or policy perspective. The Massachusetts Comprehensive Cancer Control Coalition (MCCCC) seeks to guide cancer coalitions, survivorship-focused organizations, and public health professionals to use the summit methodology to develop survivorship priorities and action plans. We describe 1) the MCCCC Survivorship Summit planning process, 2) the process of eliciting and prioritizing action plans, and 3) our evaluation results.

The MCCCC is responsible for decreasing deaths and suffering caused by cancer in the state of Massachusetts through a comprehensive approach. The MCCCC comprises more than 300 organizational and individual members. Its priorities are guided by a statewide plan (11), which was implemented by 6 working groups, including the Survivorship Working Group (SWG). Our work is funded in part by a cooperative agreement from the Centers for Disease Control and Prevention.

The goal of the SWG is to ensure that all cancer survivors in Massachusetts have equal access to information and follow-up medical, rehabilitative, and psychosocial services. The group includes 25 people from 12 organizations, representing academic, community, medical, and nonprofit voluntary sectors. Survivorship begins on the day of diagnosis and includes others who are affected by the diagnosis, including family members, friends, and caregivers (12).

## Rationale for a Cancer Survivorship Summit

Beginning in 2006, the SWG conducted a series of assessments to better understand cancer survivorship issues in Massachusetts. In the same year, questions were added to the Behavioral Risk Factor Surveillance System (BRFSS) survey in Massachusetts by the Massachusetts Department of Public Health. The BRFSS monitors disease, prevention, and quality of life through telephone interviews. BRFSS data confirmed that a substantial proportion of the state's adult population (20% of respondents aged 55 or older) had received a cancer diagnosis, with 52% of these occurring more than 5 years before the survey. Cancer survivors had similar behavioral risk factor rates (in the domains of smoking, physical activity, and weight) compared with the general population. However, rates of chronic diseases and disability were substantially higher among cancer survivors than among the general population.

The SWG then implemented a Web- and telephone-based qualitative survey to determine what barriers, issues, and concerns Massachusetts cancer survivors experienced; 1,377 survivors responded to the survey. The SWG then completed a thematic analysis to identify common experiences, which include the following: 1) the need for a central source of information on resources available in respondents' geographic area, without having to rely on support groups; 2) financial struggles that result from a cancer diagnosis, particularly among people older than 60; 3) loss of health insurance; 4) workplace discrimination; 5) lack of information about clinical trials; and 6) lack of awareness of the need for advanced care planning.

Survey participants did not broadly represent all cancer survivors. Most responses (63%) came from women, more than 97% of whom were breast cancer survivors. The survey also did not show how the common problems could be solved. The SWG determined that a statewide cancer survivorship summit was needed to develop a deeper understanding of the issues and how they could be faced through devising specific short- and long-term action steps.

## Summit Planning and Development

The Summit Planning Committee (SPC)The Summit Planning Committee (SPC), which comprised SWG members, realized that 1) specific problems faced by Massachusetts cancer survivors and their caregivers need to be clarified, and 2) the strategies to solve these problems need to be well-defined action plans. The SWG wanted the summit to include everyone involved in cancer survivorship (eg, people with newly diagnosed cancer, those living with cancer, new and long-term cancer survivors, caregivers and family members, health care professionals, public policy makers). Summit participants would be asked to define problems faced by cancer survivors and caregivers in Massachusetts and to outline potential solutions. Additionally, the summit would allow participants to interact with keynote presenters, workshop session leaders, and attendees representing health care organizations and community service organizations from around the state.

The summit planning and development process lasted from November 2006 through May 2008. SPC volunteers served in planning groups, which were chaired by the 1 or 2 people most experienced in the area. The SPC met regularly to ensure that the planning groups were functioning effectively, meeting objectives on time, and getting needed resources. Meetings occurred mostly via conference calls; informal communications occurred via e-mail and telephone. The Massachusetts Department of Public Health coordinated the planning process, organized meetings, produced and circulated meeting minutes via e-mail, and helped all planning groups.

Four planning groups were formed (Appendix). The Agenda and Content planning group developed the agenda for the summit, identified topics, and invited keynote speakers and summit working group content experts. The Sponsorship and Exhibit planning group identified potential locations, sought financial support from local and regional organizations, and invited exhibitors. The Materials and Evaluation planning group developed educational materials for the agenda to be included in summit handouts and produced and analyzed an evaluation form. The Marketing — Registration and Materials planning group developed registration materials and created and distributed summit advertisement materials.

### Summit format and action plans

The summit was a 1-day event that included keynote sessions and morning and afternoon working group sessions. Keynote speakers were 4 local and national experts, advocates, and supporters of cancer survivorship. Topics were patient navigation, advocacy, a review of the work by the MCCCC that led to the summit, and personal stories.

Ten working group session topics were identified (Table). The goal of each session was to explore the most productive ways to institute system-level change for solving problems related to each working group topic. During each session, a trained facilitator, an SWG member, first explained the charge to participants. This was followed by a 15-minute presentation by a local content area expert who described current evidence on the topic. The remainder of each session was a facilitated group discussion in which participants formulated and prioritized action plans. The desired product of each working group was a list of specific cancer survivorship problems to solve and 2 to 3 proposed system-level action plans to solve them. System-level action plans were defined as evidence-based intervention strategies, such as an organization or policy that could affect an entire system.

The Table describes the problems and action plans identified by each working group. Overall, 26 system-level action plans were identified and categorized into 5 major domains: 1) providing services within patient navigation programs; 2) offering formal training programs for providers, volunteers, and human resources; 3) providing ready access to information; 4) advocating for policy changes; and 5) developing standardized protocols and documents, such as treatment summaries and care plans.

## Summit Evaluation

At the end of the summit, participants completed a brief survey to rate their experiences. The survey assessed participants' 1) demographic characteristics, 2) role in cancer survivorship (ie, cancer survivor, health care professional, caregiver, or other support), and 3) reasons for coming to the summit. They were asked if the summit met their expectations, if their voices were heard, and if the summit helped them advocate for cancer survivorship issues. Participants also rated each summit session and the summit overall.

### Analysis of participants

In total, 220 participants attended the summit, 144 of whom (65%) completed an evaluation form. Most were female (93%), aged 50 or older (64%), and non-Hispanic white (85%); approximately 11% of participants were non-Hispanic black. Approximately 61% of participants identified themselves as cancer survivors, and 66% indicated that they attended the summit in a professional role (eg, clinician, researcher, professional cancer advocate). Approximately one-third of participants (34%) were both cancer survivors and professionals.

The most common reasons that participants attended the summit were wanting to learn more about cancer survivorship issues (65%), having an interest in a specific topic on the summit agenda (55%), wanting to network (48%), and wanting to make a difference (42%).

### Evaluation results

Participant ratings of the summit were positive. The majority reported the following: the summit met their expectations (90%), there was opportunity for their voices to be heard (81%), and attending the summit would better help them advocate for cancer survivorship issues (84%). The strategies chosen at the working group sessions were rated excellent or very good (81% for the morning session and 76% for the afternoon session). Eighty-nine percent rated the summit overall as excellent or very good.

Ratings of the summit did not vary by participant demographics. However, there were differences according to professional versus nonprofessional status. Compared with professionals, nonprofessionals were less likely to 1) say they were extremely satisfied with the strategies decided on in the morning session (56% vs 30%, *P* = .006) and in the afternoon session (65% vs 40%, *P* = .004), 2) rate as excellent the extent to which the summit met their expectations (44% vs 29%, *P* = .05), 3) rate as excellent the extent to which the summit would help them advocate for cancer survivorship issues (43% vs 17%, *P * = .003), and 4) rate the summit overall as excellent (60% vs 42%, *P* = .05). There were no differences between the groups in reporting as excellent the extent to which they believed their voices were heard.

The SWG used frequency distributions to describe each variable and compared each of the 6 summit ratings by participant characteristics. Each rating variable was dichotomized to compare participants who rated variables excellent/extremely satisfied with those who did not. Cross-tabulations were computed and Fisher exact tests were used to assess significant differences.

## Discussion

A survivorship summit is a feasible, effective way to enlist community input to drive the mission, strategic planning efforts, and priorities of cancer coalition and advocacy groups. On the basis of the usefulness of the action plans identified, the results of the evaluation, and the perceptions of the staff, the MCCCC Survivorship Summit was a success. The summit ran smoothly, and the overall format was well received. Only 1 working group session was cancelled. In their survey evaluations, some participants expressed the need for longer discussion periods during the work group sessions. For example, because of limited time, the quality-of-life work group discussion focused mainly on pain.

The system-level action plans reflect the need for strategies to 1) develop patient navigation programs that meet the needs of cancer survivors; 2) improve access to information and services for cancer survivors and caregivers; 3) train health care and other service providers and volunteers to help cancer survivors and caregivers during all phases of survivorship, including posttreatment phases; 4) advocate for policy changes; and 5) develop standardized protocols and documents (eg, treatment summaries and care plans). The priority areas include changes in policies that highlight the importance of advocacy and the need for training in this area.

Several lessons learned can improve the success of future similar efforts. Sufficient resources and highly committed personnel are necessary to help develop each component and to conduct activities at the summit. Investment of coalition members is essential, and a planning period of at least 1 year is required. Adequate personnel are needed particularly at crunch time to ensure the smooth running of summit plans. In the MCCCC summit, graduate students were available to assist with the workload through the academic affiliations of some summit planners.

Achieving geographic representation from across the state was difficult, even though Massachusetts is a small state and the summit was near a major highway. Most participants were from within the county where the summit was held or the 3 adjacent counties. Participants from more distant counties were more likely to be professionals than nonprofessionals. To achieve geographic representation, summit planning efforts should include finding resources to pay for transportation for people who are not geographically close and coordinating with local organizations and advocacy groups to promote options (eg, chartering buses, carpooling). In larger states, regional summits may be more appropriate to assure that different geographic regions are represented.

The summit was not as well received by nonprofessionals as it was by professionals. We may not have adequately communicated the purpose of the summit. The goal was for participants to identify coalition priorities, not to provide information or resources. To prevent misunderstandings, all correspondence regarding such a summit should include clear objectives for the summit, state what the expectations are for participants, and pilot test these materials with the intended audience.

The evaluation protocol for this summit has limitations. Evaluation was conducted only immediately after the summit concluded. Longer-term evaluation of the summit's effects on attendees would have been useful. For the SWG, the summit has been translated into long-term work plans, but no separate evaluation of effects of the identified action plans on the work and advocacy priorities of SWG members were conducted.

The MCCCC Survivorship Summit can guide future efforts of this coalition. The summit also allowed the coalition to recruit additional members; attendees who volunteered to work on any action plans were noted during the summit and will be contacted to participate in the implementation of these plans. After the summit, action plans were disseminated to all summit participants via e-mail and mail. During the next 5 years, the SWG will implement these action plans across Massachusetts.

## Acknowledgments

Funding for the MCCCC Survivorship Summit was provided in part by a cooperative agreement from Centers for Disease Control and Prevention, National Cancer Prevention and Control Program grant to the Massachusetts Comprehensive Cancer Prevention and Control Program. Funding was also provided by the American Cancer Society, Massachusetts Department of Public Health Women's Health Network and Men's Health Partnership and Bureau of Communicable Disease Control, Bayer HealthCare, Dana-Farber Cancer Institute, Lance Armstrong Foundation, Massachusetts Cancer Prevention Community Research Network, New England Coalition for Cancer Survivorship, and Pfizer Oncology.

We thank Snaltze Pierre, coordinator of the MCCCC, for assistance in coordinating work of the SWG and the SPC. We thank the other members of the SPC for implementing this program, including Janice McGrath, SWG cochair; Zana Baruch; Cindy Blood; Lila Brady; Diane Cotting; Carol Curtiss; Kathy Hart; Lynne Graziano Morin; Marcie Saganov; and Palmira Santos. We thank Green Associates, LLC, for assistance in coordinating summit planning and implementation. We thank Joyce Kulhawik, Harold P. Freeman, Palmira Santos, Harriet Chandler, and Susan Quinones for participating as keynote speakers. We thank Seana Quental, Sarah Hunt, and Italo Brown for their assistance with preparing and implementing activities; Andrew Prout for his technical assistance; Jane Hilbert Davis for training facilitators; the volunteers who served as local content experts, working group facilitators, and recorders; and summit participants.

## Figures and Tables

**Table.** Working Group Session Topics and Action Plans Identified, Massachusetts Comprehensive Cancer Control Coalition (MCCCC) Survivorship Summit, Massachusetts, 2008

**Session**	**Problem**	**Action Plans**
Finding balance: caring for a loved one and yourself	To decrease hardships for cancer caregivers	Place computers in cancer treatment center waiting areas to ensure that caregivers have access to specialized, credible, user-friendly information. Advocate for a standard "caregiver bill of rights." Create a checklist of people who need to be seen or consulted after a person receives a diagnosis.
Addressing financial and legal issues	To alleviate the financial weight of cancer and its specific legal issues	Work with hospitals to develop quality improvement projects that focus on the financial components of delivery of care such as authorizations, referral processes, and billing issues. Lobby to rescind the 5-month waiting period for Social Security benefits for terminal cancer patients. Lobby legislators to reevaluate the Medicare Part D pharmaceutical benefit to eliminate the coverage gap.
Workplace discrimination: protect yourself	To address workplace discrimination, areas of advocacy, and productive ways to institute change	Develop programs in which independent specialists in the area of workplace discrimination conduct formal training of human resources staff and supervisors at worksites through a variety of media (eg, presentations, interactive Web sites, and pamphlets). Develop educational programs that focus on workplace discrimination specifically against cancer survivors, empowering employees with information on how to handle such discrimination.
Saving lives through clinical trials	To increase access to and participation in clinical trials	Make available informed consent forms in multiple languages. Create a DVD to distribute information on available clinical trials. Implement patient navigation program components that address transportation, information delivery, and support systems for people recruited for and participating in clinical trials.
Improving quality of life: pain and symptom management	To improve pain and symptom management	Implement a media advocacy program or marketing campaign to increase awareness on cancer pain and quality of life. Develop standard training programs for health care professionals to communicate with patients regarding all symptoms patients might experience. Incorporate training into patient navigation programs to address quality of life and long-term plans. Advocate for legislation to mandate that insurance companies include alternative/complementary medicine options as covered benefits.
Connecting with needed resources	To connect cancer patients and their loved ones with resources in their communities	Advocate for legislation mandating patient navigation programs at health care facilities. Ensure that patient navigators receive comprehensive training on all aspects of cancer survivorship (including medical, psychosocial, and financial issues) so that they will be fully equipped to provide survivors and caregivers with needed information on all available resources, both locally and nationwide. Develop systems to connect young survivors (younger than age 40) to existing resources both locally and nationally that address issues such as posttreatment side effects and impact of treatment on their finances, careers, and fertility.
Caring for cancer survivors: victims of success?	To provide survivors and their caregivers with information about managing the late effects of cancer treatment	Develop treatment summary and care plans. Develop automated electronic medical record templates that summarize treatment protocols and care plans.
Coping with intimacy and relationships: sexuality and psychosocial issues	To improve information and assistance concerning intimacy and relationship issues	Establish CME-credited Web-based training sessions for providers on issues of sexuality among cancer survivors. Include sexuality issues and options on survivorship care plans.
Navigating the challenges of the health care system: patient navigation.	To enhance the role of patient navigation as a method to address challenges in the health care system	Institute a broad system of patient navigators and develop curricula that support patient navigation through the Department of Public Health. Establish guidelines and best practices for patient navigation by government and public agencies.
Volunteering: healing ourselves and helping others	To make volunteering opportunities more available to cancer survivors	Create a volunteer database that matches volunteers with positions. Establish a universal training course for volunteers to ensure that proper standards are used.

Abbreviation: MCCCC, Massachusetts Comprehensive Cancer Control Coalition.

## Appendix. Description of Planning Groups for the MCCCC Survivorship Summit

### Agenda and content group

- Identified working group session topics for unmet needs of cancer survivors based on Web-survey findings and scientific literature. Determined working group session format.
- Identified and invited keynote speakers. Factors considered included person's contributions to cancer survivorship-related work; relevance of this work to summit goals; and ability of the speaker to be motivational, inspiring, and knowledgeable. Speakers were informed of the summit goal, specific objectives, and expected products. Confirmation of speakers required discussions related to the topic of presentations, honoraria, accommodations needed, and travel.
- Identified and invited working group content experts, facilitators, and recorders. Content experts were selected on the basis of their area of expertise and the breadth of their knowledge and understanding in the content areas. Facilitators and recorders were mostly MCCCC members and students at a local school of public health.
- Developed final summit agenda, including start and finish times, order and length, and sessions and breaks.
- Trained workshop facilitators and briefed recorders. The goal of this training was to provide facilitators with facilitation skills, including establishing ground rules for working group discussions such as the importance of participating fully (without any side conversations) and how to deal with difficult participants without distracting focus of the group during the discussion. Training included didactic lecture, role-playing possible scenarios, and discussions on how to prioritize problems and action plans proposed by participants.
- Applied for and gained approval for continuing medical education (CME)/continuing education unit (CEU) credits for summit participants. These applications were made through the Office of Continuing Medical Education of the Massachusetts Department of Public Health, Bureau of Communicable Disease Control.
- Identified equipment and technical support needs and developed plans to meet these needs.

### Sponsorship and Exhibit Group

- Developed sponsorship packet, including letter of invitation/solicitation to sponsors.
- Developed sponsorship guidelines, made available on sponsorship brochure. Levels of sponsorship ranged from $500 or less to $20,000 or more. For example, those sponsoring $20,000 or more were given a prominent listing in the summit program, including their logo, acknowledgment of their sponsorship during the opening session for the summit, a link to their Web site from the summit Web site, acknowledgement of their sponsorship in press materials, a complimentary table in the exhibit area, and complimentary summit registration for 6 people. The $500 sponsorship included a listing in the summit program and a complimentary summit registration for 1 person.
- Identified and pursued potential summit sponsors.
- Researched and identified suitable venues. Factors considered were cost, facility size, number of breakout rooms, and location.

### Materials and Evaluation Group

- Prepared materials for summit participants, which were made available on the summit Web site and in handouts distributed at the summit, including evidence-based summaries of the current state of understanding of the 10 working group session topics.
- Developed summit evaluation survey.
- Analyzed summit evaluation data. Presented findings to the MCCCC Survivorship Committee. Distributed results as part of the summit's final report.

### Marketing — Registration and Materials Group

- Designed summit "Save the Date" card (both e-mail and hard copy version) and summit brochure.
- Developed summit marketing plans, mailed summit brochures to 20,000 potential participants, sent e-mail blasts, posted on Web site, and advertised in publications and radio.
- Engaged members of the MCCCC to help with summit promotion. Marketing activities were targeted toward cancer survivors and their loved ones, cancer health care providers, cancer centers, clinics, hospitals, and nonprofit cancer-related organizations.
- Collected and monitored registration forms.
- Established a minimum registration cost ($20) to ensure commitment from participants. Provided materials for scholarship on request to cover registration cost.

## References

[1] Statistics for 2009. American Cancer Society.

[2] National Action Plan for Cancer Survivorship. Centers for Disease Prevention and Control and Prevention.

[3] Alfano CM, Rowland JH (2006). Recovery issues in cancer survivorship: a new challenge for supportive care. Cancer J.

[4] McCabe MS, Jacobs L (2008). Survivorship care: models and programs. Semin Oncol Nurs.

[5] Hewitt M, Greenfield S, Stovall E (2005). From cancer patient to cancer survivor: lost in transition. From cancer patient to cancer survivor: lost in transition.

[6] Meyerowitz BE, Kurita K, D'Orazio LM (2008). The psychological and emotional fallout of cancer and its treatment. Cancer J.

[7] Taskila T, Lindbohm ML (2007). Factors affecting cancer survivors' employment and work ability. Acta Oncol.

[8] Stanton AL (2006). Psychosocial concerns and interventions for cancer survivors. J Clin Oncol.

[9] Ganz PA (2001). Late effects of cancer and its treatment. Semin Oncol Nurs.

[10] Fosså SD, Vassilopoulou-Sellin R, Dahl AA (2008). Long term physical sequelae after adult-onset cancer. J Cancer Surviv.

[11] Massachusetts statewide plan to reduce and eliminate death and suffering caused by cancer 2006-2011. Massachusetts Comprehensive Cancer Control Coalition.

[12] About cancer survivorship research: survivorship definitions, National Cancer Institute.

